# Beyond DNA Damage: Exploring the Immunomodulatory Effects of Cyclophosphamide in Multiple Myeloma

**DOI:** 10.1097/HS9.0000000000000350

**Published:** 2020-04-03

**Authors:** Dawn Swan, Mark Gurney, Janusz Krawczyk, Aideen E. Ryan, Michael O’Dwyer

**Affiliations:** 1 Department of Hematology, University Hospital Galway, Galway, Ireland; 2 School of Medicine, National University of Ireland Galway, Galway, Ireland; 3 Discipline of Pharmacology & Therapeutics, School of Medicine, College of Medicine Nursing and Health Sciences, National University of Ireland Galway, Galway, Ireland; 4 Regenerative Medicine Institute, School of Medicine, College of Medicine Nursing and Health Sciences, National University of Ireland Galway, Galway, Ireland.

## Abstract

The alkylating agent cyclophosphamide has been used in the treatment of multiple myeloma for over 60 years. At low doses, cyclophosphamide also has significant immunomodulatory activity, which can be used to modify the immunosuppressive tumor microenvironment in order to augment responses to existing therapies. Immune-mediated therapies are becoming more widespread in modern approaches to myeloma treatment. In this review, we discuss the effects cyclophosphamide has on the immune system, and how it can be used synergistically with other treatment modalities including the immunomodulatory agents, monoclonal antibodies and cellular therapies.

## Introduction

Cyclophosphamide is a member of the oxazaphosphorine family of mustard-alkylating agents. It has been used in the treatment of malignant conditions, including multiple myeloma (MM), since its discovery in 1958.^1^

Cyclophosphamide has several mechanisms of action, partly dependent upon the dose of the drug being utilized. At high doses it acts as an alkylating agent, mediating its cytotoxicity through DNA damage, however at low doses it has immunomodulatory effects (reviewed in^2^). Definitions of low and high doses are not standardized between clinical trials. Low dose cyclophosphamide is reported as referring to a single dose of 1 to 3 mg/kg, whereas high-dose may mean values of 120 mg/kg up to several grams/kg.^3^ Metronomic dosing describes iterative low doses of oral cyclophosphamide, often 50 mg daily or 100 mg every other day.^4^

Cyclophosphamide itself is a prodrug, hydrolyzed in the liver by cytochrome P450 enzymes (predominantly CYP 2B6 and 3A4)^5^ into 4-hydroxycyclophosphamide and its tautomer aldophosphamide,^6,7^ which are taken up by target cells by passive diffusion and active transport via P-glycoproteins.^8^ Once in the cytoplasm, aldophosphamide is converted into the active products acrolein and phosphoramide mustard. Both acrolein and phosphoramide mustard are alkylating agents, producing DNA strand breaks. Phosphoramide mustard also causes DNA cross-linking, which leads to cellular necrosis or apoptosis, and likely accounts for a greater proportion of cyclophosphamide's cytotoxicity than its alkylating effect.^9^ These processes are regulated by aldehyde dehydrogenase (ALDH) 1, which converts aldophosphamide into non-toxic carboxyphosphamide, and the anti-oxidant glutathione (GSH), which forms stable conjugates with acrolein and phosphoramide mustard.^10–12^

Cyclophosphamide has also been used in the mobilization of stem cells for apheresis and peripheral blood collection for several decades. At very high doses, cyclophosphamide triggers release of proteases which cleave bone marrow adhesion molecules, such as vascular cell adhesion molecule-1 (VCAM-1) and C-X-C chemokine receptor type 4 (CXCR4), facilitating release of hematopoietic stem cells from the bone marrow niche into the peripheral blood.^13,14^

In addition to its ability to damage cellular DNA, cyclophosphamide also has significant immunomodulatory activity, affecting several classes of immune cells. Activated immune cells kill tumor cells specifically, avoiding some of the toxicities of traditional chemotherapy, can overcome drug resistance^15^ and have memory, enabling continued tumor surveillance (reviewed in^16^). These effects are evident at low doses. This was demonstrated in a murine cancer model, in which tumor cells were injected subcutaneously into the flanks of mice allowing formation of measurable tumor masses. Reduction in tumor volume following administration of low dose cyclophosphamide was only seen in immune-competent mice, whereas high doses produced responses in both immune-competent and nude mice.^17^

The ability of MM cells to circumvent immune-detection through interactions with the immunosuppressive tumor microenvironment (TME), and the progressive decline in immune function seen in these patients is well described (reviewed in^18^). There are many novel anti-MM therapies available or in clinical development including monoclonal antibodies and cellular therapies, which rely upon an intact immune system for efficacy. The immunomodulatory activities of cyclophosphamide could therefore be employed to switch the TME from an immunosuppressive to immunostimulatory environment, synergizing with these newer agents in order to augment their activities.

**Figure 1:**
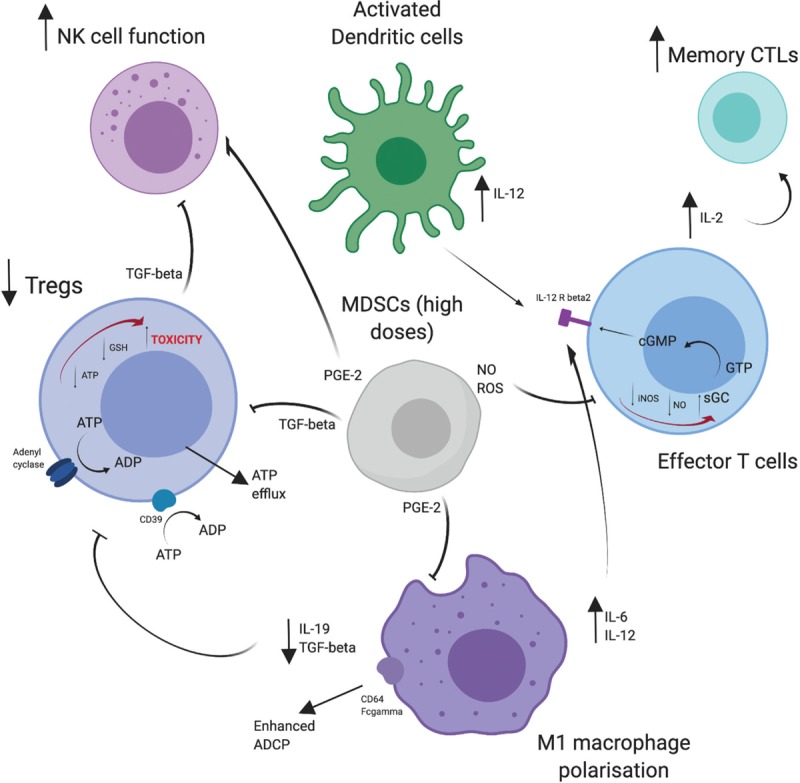
The immunomodulatory effects of low-dose cyclophosphamide in MM.

In this review, we focus upon the immunomodulatory actions of cyclophosphamide. We first describe various critical cellular components of the TME and the effect that cyclophosphamide has upon them (summarized in Fig. 1), and secondly, the clinical impact and current role of cyclophosphamide in modern MM treatments.

## Immunomodulatory effects

The tumor microenvironment (TME) is comprised of numerous cellular subsets, with both immunostimulatory and immunosuppressive cells present. The role of these subtypes in MM, and how their activities are affected by cyclophosphamide is discussed in the following section.

### Regulatory T cells (Tregs)

Tregs are an immunosuppressive subset of T-lymphocytes, characterized by CD4 and Foxp3 positivity, whose primary function is to enable tolerance to self-antigens and prevent development of autoimmune reactions by suppressing both innate and adaptive immune functions. In particular, high affinity antigen-specific cytotoxic T-cells and memory cells are impaired.^19^ Tregs are known to be increased in patients with MM and monoclonal gammopathy of uncertain significance (MGUS), enabling immune evasion and facilitating disease progression, although some inconsistent associations with disease progression have been reported. For example, one study of approximately 200 patients with MGUS or MM found that patients with ≥5% Tregs had a statistically significantly reduced time to progression than those with lower Treg levels. However, these findings were not replicated in a smaller study of 10 patients with MGUS or MM and 5 healthy donors.^20,21^ MM cells have been shown to drive expansion and activation of Tregs through interferon-1 release.^22^

The effect of cyclophosphamide on Tregs was first observed in a study performed in 1974, before Tregs themselves had been identified. Topical administration of 2,4-dinitrofluorobenzene (DNFB) is used to induce contact sensitivity in animal models. In this study, dinitrobenzene-sulphonic acid sodium salt (DNBSO_3_) was injected intravenously to induce tolerance to subsequent DNFB application, preventing a skin reaction from developing. This effect could be abrogated by giving cyclophosphamide 3 days prior to contact sensitization. This was shown to be associated with increased proliferation of T cells, thought to be due to reduction in levels of a suppressive cellular subset by the cyclophosphamide. Transfer of lymph node cells from sensitized animals also reduced subsequent reactions in non-sensitized animals, by transferring the yet to be identified, immune-suppressive Tregs.^23^ Further work identified a cyclophosphamide-sensitive T-cell population, which could suppress antigen-specific cytotoxic T-cell lymphocytes (CTLs) in a mouse model.^24^ In a T cell-deficient L5178Y cyclophosphamide-resistant lymphoma murine model, the combination of 150 mg/kg cyclophosphamide with transfer of tumor-specific immune cells produced tumor regression, whereas cyclophosphamide or immune cells alone had no effect, thus showing that cyclophosphamide was able to eliminate suppressor T-cells enabling activity of tumor-sensitized CTLs.^25^

Susceptibility of Tregs to cyclophosphamide is thought to be due to their relative depletion of intracellular ATP compared with effector T cells. This is due to expression of high levels of CD39, which converts extracellular ATP to ADP, generating an ATP sink and stimulating efflux of intracellular ATP, alongside low levels of microRNA (miRNA)-142-3p, which inhibits conversion of intracellular ATP to cyclic AMP.^26^ Reduced levels of ATP lead to impaired production of GSH, required to neutralize the toxic products of cyclophosphamide. To compound matters, Tregs have defective DNA repair mechanisms compared with effector T cells, increasing susceptibility to the DNA cross-linking effects of cyclophosphamide.^27^ Low dose cyclophosphamide may also inhibit the suppressive function of Tregs. Intraperitoneal administration in a murine model impaired proliferative capacity, associated with downregulated expression of the glucocorticoid-induced TNFR family-related gene (GITR), which acts as a costimulatory molecule to enhance Treg proliferation.^28^

Schedule and dose of cyclophosphamide has an impact on Treg function. Continuous daily administration may lead to drug-resistance and impaired immunomodulation. For example, in one study patients with breast cancer were given 50 mg cyclophosphamide twice daily on alternating weeks. Reduced Treg numbers and function were seen. However in another study in advanced cancer patients, in which individuals received 50 mg daily for approximately 3 months, the proportion of Tregs was reduced, but not their functional capabilities^29^ (4). In an animal model, a 6 day drug-free period was reported to produce sustained CTL levels compared with 9 or 12 day intervals.^30^

In summary, Tregs are an immunosuppressive T-cell subtype, which are enriched in patients with MM, and have been linked to immune evasion and disease progression. Compared with other T lymphocytes, they are particularly sensitive to cyclophosphamide-mediated killing, as a consequence of low levels of intracellular ATP and impaired DNA repair mechanisms.

### Effector T cells

CD8 expressing T cells recognize antigen displayed by MHC Class I (major histocompatibility complex) present on the majority of nucleated cells. Once activated they release the contents of their cytotoxic granules leading to cellular apoptosis. Profound effector T cell dysfunction occurs in MM. Tumor-specific T cells expansions are more commonly seen in patients with a low tumor-burden, in monoclonal gammopathy of uncertain significance (MGUS), the pre-malignant form of MM, and in those with prolonged survival.^31^ Moreover, exposure of tumor-specific T cell populations from MGUS patients to autologous malignant cells is associated with robust production of cytokines, whereas this is not seen using T-cells from MM patients, suggesting impaired functionality with disease progression.^32^

Low dose cyclophosphamide has been shown to improve T-cell responses to T cell receptor (TCR) stimulation and enhance production of tumor antigen-specific T cells in cancer patients. This is partly, but not entirely due to the reduction in Treg-mediated immune suppression.^4,29^ Low dose cyclophosphamide has been demonstrated to skew T helper cells from a Th2 profile to a Th1 profile, characterized by secretion of IL-2, which stimulates expansion of memory CTLs.^33^ This may be partly attributable to inhibition of inducible nitric oxide synthase (iNOS), required for production of nitric oxide (NO). Low levels of NO activate soluble guanylyl cyclase (sGC), which produces 3’,5’-cyclic guanosine monophosphate (cGMP) from guanosine-5-triphosphate (GTP). cGMP induces expression of IL-12 receptor β2, thereby promoting IL-12-dependent Th1 polarization of helper T cells.^34,35^ Increased levels of IL-17 producing CD4+ helper T-cells have also been identified following cyclophosphamide exposure. Presence of higher levels of these Th17 cells, alongside low levels of Tregs has been associated with improved survival in patients with MM.^31^ Interestingly, intestinal bacteria (particularly gram-positive *Lactobacilli johnsonii* and *Enterococcus hirae*) may have a role to play in this observation. In a murine experiment, following administration of a low dose of cyclophosphamide, intestinal permeability developed, and bacteria were seen to translocate to lymph nodes, wherein they stimulated Th1 and Th17 immune responses. Addition of the glycopeptide antibiotic vancomycin inhibited this effect,^36^ although the mechanism underpinning cyclophosphamide-induced intestinal permeability has not yet been identified.

Failure to mount an effective adaptive immune response is a common mechanism of immune evasion across cancer subtypes. Immunogenic cell death (ICD) describes a form of apoptosis capable of inciting an adaptive immune response against pathogen or cancer-derived antigens. Cytotoxic agents vary in their tendency to favor an immunogenic form of regulated cell death. The mechanisms underlying ICD are well understood and have been reviewed previously.^37^ Briefly, the endoplasmic reticulum (ER) stress response is a key initiating factor. ER stress occurs when there is an excess of unfolded or misfolded proteins within a cell.^38^ MM cells produce high levels of intracellular immunoglobulins resulting in high levels of ER stress. Compensatory mechanisms lead to cell-surface translocation of calreticulin, which acts as an ‘eat me’ signal, stimulating phagocytosis and dendritic cell activity, and ultimately leading to enhanced activation of tumor-specific cytotoxic T cells. Danger-associated molecular patterns (DAMPs) act as required adjuncts to this process in the context of malignancy. While definitive evidence for this mechanism contributing to the in vivo responses to cyclophosphamide observed in multiple myeloma is lacking, cyclophosphamide has been shown to induce ER stress and hallmarks of ICD in high grade lymphoma and mouse models of thymoma.^33,39^ Given the reliance of myeloma cells on ER stress pathways, ICD and subsequent antigen presentation to the adaptive immune system is postulated as an important initiating step in a cascade of immunomodulating effects attributable to cyclophosphamide,^40,41^ although this area warrants further study.

In summary, effector T cell responses are diminished in MM patients. Low-dose cyclophosphamide improves tumor-specific T cell activity by reducing Treg number and function, skewing T helper cells from a Th2 to Th1 phenotype, increasing Th17 numbers, and inducing ICD.

### Dendritic cells (DCs)

DCs are professional antigen presenting cells (APCs) which provide a crucial bridge between the innate and adaptive arms of the immune system. High circulating levels of IL-6 in myeloma patients has been shown to impair generation and function of DCs, stimulating CD34+ cells to differentiate into monocytic cells which can perform phagocytosis but are unable to present myeloma epitopes to, and thereby activate, T cells.^42^ Additionally, there are 2 dominant subsets of DCs- myeloid and plasmacytoid. Plasmacytoid DCs are increased in the BM of MM patients and may actually promote MM cell growth, survival and proliferation.^43^ These plasmacytoid DCs express high levels of PD-L1 (programmed death- ligand 1) causing T-cell inhibition.^44^

DCs isolated from mice treated with cyclophosphamide induced more potent allogeneic and antigen-specific proliferation of T cells compared with those from unexposed mice, and had higher levels of IL-12 secretion.^45^ Use of cyclophosphamide to augment responses to DC vaccine-based immunotherapies has provided positive results in murine models,^46^ and in early stage clinical trials in the context of renal cell cancer.^47^

In summary, DC function is altered in MM patients, leading to reduced T cell activation, which can be abrogated by the addition of cyclophosphamide.

### Macrophages

Macrophages affect disease growth and progression in MM and other cancers. Tumor-associated macrophages (TAM) are derived by recruitment and activation of circulating monocytes by cytokines and chemokines produced by tumor cells and bone marrow stromal cells (BMSCs). Activated macrophages are polarized with either an M1 or M2 phenotype. M1 macrophages are pro-inflammatory and produce high levels of TNF-α and IL-12, often in response to infections. TAMs more commonly resemble M2 macrophages, which have immunosuppressive activity, and stimulate angiogenesis favoring tumor growth,^48,49^ providing pro-tumorigenic signaling. Increased levels of M2 macrophages have been seen in MM patients with progressive disease compared to those in remission.^50^ In *vitro* studies have shown that IL-12, typically produced by M1 rather than M2 macrophages can downregulate myeloma cell angiogenesis, and in a mouse model, can impair tumor growth following injection with multiple myeloma cell lines.^51^ The role of IL-12 in promoting development of Th1 helper T cells, which stimulate expansion of memory CTLs has been described above.^52^ Additionally, production of IL-2, interferon-gamma and TNF-β secreted by Th1 cells also activate macrophages.^53^ Moreover, predominance of M2 macrophages has been linked to resistance to combination therapies including the anti-CD38 monoclonal antibody daratumumab, alongside immunomodulatory agents such as lenalidomide.^54^

One group analyzed macrophage phenotype and function following administration of cyclophosphamide 50 mg/kg in a murine model. These macrophages showed increased production of pro-inflammatory IL-6 and IL-12 associated with the M1 phenotype, and reduced levels of anti-inflammatory IL-19 and TGF-β,^55^ which have been shown to induce immune-suppressive Tregs.^56^

A study by Pallasch *et al* using a malignant B-cell line, resistant to the CD52 monoclonal antibody alemtuzumab, showed that secretion of prostaglandin E2 (PGE2) by the malignant B-cells inhibited macrophage-mediated phagocytosis. The combination of cyclophosphamide and alemtuzumab showed synergism, leading to almost complete elimination of the malignant cells, which did not occur using other alkylating agents. Exposure of the cell line to cyclophosphamide induced an ‘acute secretory activation phenotype’ (ASAP), characterized by production of tumor necrosis factor-α (TNF-α) and vascular endothelial growth factor A (VEGF A) by the B-cells, alongside reduction in expression of macrophage-suppressive PGE2.^57^ Exposure of MM cells to low-dose cyclophosphamide has similarly been shown to induce a secretory response, leading to enhanced macrophage-mediated antibody dependent cellular phagocytosis (ADCP) in daratumumab-treated MM cells, both *in vitro* and in a phase 1b clinical trial of upfront daratumumab with cyclophosphamide, bortezomib and dexamethasone in transplant eligible patients (NCT02951819). Cyclophosphamide-conditioned macrophages were found to have increased levels of CD64 Fc gamma receptor expression, required for ADCP, whereas MM cells had reduced levels of the ‘don’t eat me’ antigen CD47, possibly further enhancing phagocytosis. Additionally, MM cell surface expression of SLAM-F7 was increased, suggesting a possible synergy with elotuzumab (anti-SLAM-F7 monoclonal antibody).^58^

In summary, TAMs with an anti-inflammatory M2 phenotype are seen at increased levels in MM patients with progressive disease. Low-dose cyclophosphamide induces an acute secretory response from MM cells leading to enhanced anti-tumor phagocytic activity.

### Myeloid derived suppressor cells (MDSCs)

MDSCs are a heterogenous group of CD33 positive myeloid-derived cells, which are typically CD14 positive monocytic MDSCs or CD14 negative granulocytic MDSCs,^59^ the latter being more prevalent in MM patients.^60^ These cells have numerous immunosuppressive activities, facilitating tumor survival. Effector T cells are impaired through depletion of arginine, production of nitric oxide and reactive oxygen species.^61–63^ MDSCs produce PGE2, suppressing macrophage phagocytosis.^64^ They promote expansion of immunosuppressive Tregs through TGF-β-dependent and independent mechanisms,^65^ and also cause NK cell energy via TGF-β signaling and PGE2.^66,67^ Despites its many modes of enhancing immune function in myeloma, cyclophosphamide has also been noted to induce MDSCs, leading to impaired T-cell anti-tumor responses.^68,69^ However, in a murine model, this was seen using 100–300 mg/kg but not smaller doses (10–40 mg/kg). The authors hypothesized that cytokine release in response to leucodepletion could be contributary, and perhaps this does not occur with lower doses.^70^

In summary, MDSCs have several immunosuppressive functions. They may be induced by high levels of cyclophosphamide, however this has not been noted using smaller doses.

### Natural killer (NK) cells

NK cells are a critical component of the innate immune system. Their activity is regulated by a fine balance between signals produced by inhibitory and activating NK receptors, which recognize ligands expressed by tumor cells or virally-infected cells. NK cellular function is impaired in MM by a number of mechanisms. TGF-β, produced by MM cells and Tregs, downregulates NK-activating receptors and impairs cytotoxicity.^71^ MM cells produce IL-6, which inhibits NK cell function, and PGE2 from MDSCs also inhibits NK activation via the natural cytotoxicity regulators (NCR), NKG2D and CD16/ FcγRIIIA receptors (reviewed in^72^) (18). A study in 9 chemotherapy-resistant cancer patients, who received metronomic cyclophosphamide at 50 mg twice daily on alternate weeks reported a reduction in absolute numbers of circulating Tregs after 30 days, and a corresponding increase in NK cytotoxicity, which improved to levels not significantly lower than those recorded in healthy donor controls. The remaining Tregs also appeared to lose their NK inhibitory capacity, as selective depletion of this population did not further improve NK function.^4^ In their study, Pallasch et al also showed that pre-incubation of NK cells with conditioned media from cyclophosphamide-treated leukemia cells significantly improved alemtuzumab-induced NK-mediated ADCC. ADCC was significantly reduced in the presence of PGE2, and conversely, significantly enhanced by the addition of VEGF or TNF-α, produced by the leukemia cells in response to cyclophosphamide.^57^

In summary, NK cells are impaired in MM patients by production of TGF-β by Tregs, IL-6 from MM cells and PGE2 from MDSCs. Cyclophosphamide improves NK cell function by reducing the prevalence of Tregs, any by causing reduced production of PGE2 and increased secretion of proinflammatory cytokines.

## Immunomodulatory role within MM treatment regimens – synergy with immune-mediated therapies

Low doses of cyclophosphamide, given more frequently, were first observed to have surprising activity in relapsed refractory myeloma patients several decades ago. A small, 20-patient study, employed a regimen of weekly cyclophosphamide (150–300 mg/m^2^) with alternate day prednisolone. Overall response rate (ORR) was 50%, which included 3 patients who had failed previous cyclophosphamide-based regimens in which the drug was given at larger, less frequent doses.^73^ Metronomic cyclophosphamide is also a very well tolerated option for patients. A combination of low-dose cyclophosphamide (50 mg daily) with prednisolone (15 mg daily) was used in 27 relapsed patients with significant co-morbidities, precluding the use of more intensive therapy. This included individuals with dialysis-dependent renal failure, severe infections during previous therapies which led to treatment discontinuation, and severe cardiac failure. Such patients have limited therapeutic options and are normally poorly represented in clinical trials. ORR was 67% and adverse events were generally mild. At a median follow-up of 11 months, responding patients had a median OS of 22 months and PFS had not been reached.^74^ Even in the era of novel therapies, the activity of metronomic cyclophosphamide in combination with a steroid has surprised clinicians and researchers. The FOCUS study was a randomized phase 3 study of single agent carfilzomib versus low-dose steroids in relapsed refractory multiple myeloma (RRMM). The study protocol gave the option of adding metronomic cyclophosphamide (50 mg daily) to the steroid control arm, which was chosen by 95% of patients. Median OS was 10.2 months versus 10 months and median PFS was 3.7 months versus 3.3 months for single-agent carfilzomib and steroids +/− cyclophosphamide respectively. The study failed to meet its primary end-point because the control arm performed far better than expected, even compared with a highly active drug.^75^

Synergy is seen between low doses of cyclophosphamide and steroids, which is enhanced by the addition of further immune-mediated therapies, as described in the following section.

### Immunomodulatory agents (IMiDs)

Low doses of cyclophosphamide can synergize with immunomodulatory agents, with the capability to produce responses in previously resistant patients. A phase 1/2 trial tested daily metronomic cyclophosphamide with lenalidomide and dexamethasone (REPEAT study) in patients with lenalidomide-resistant disease. ORR was 67%, with progression free survival (PFS) of 12 months and overall survival (OS) 29 months.^76^ Results were similar in all subsets, including patients with resistance to both lenalidomide and bortezomib, and those with adverse cytogenetic risk profiles. IMiDs exert their anti-myeloma effect through binding to the ubiquitin ligase enzyme cereblon, promoting ubiquitination and proteasome-mediated degredation of the IKZF transcription factors Ikaros and Aiolos. Reduced baseline expression of cereblon has been associated with poor responses to lenalidomide. Analysis of samples from the REPEAT study found reduction in cereblon expression and elevated c-myc levels in patients at the time of acquisition of lenalidomide resistance.^77^ Review of peripheral blood immune cell subsets in these patients revealed a significant mid-cycle decrease in the cereblon substrate proteins Ikaros and Aiolos alongside an increase in T-cell activation, which fell back to baseline after 1 week of lenalidomide interruption. In *vitro*, enhanced peripheral blood mononuclear cell-mediated killing of both lenalidomide-sensitive and resistant MM cells was observed, providing further evidence for the synergistic effect of cyclophosphamide when added to lenalidomide and dexamethasone even in patients with lenalidomide resistance.^78^

A multicenter study reviewed outcomes in 31 patients receiving lenalidomide and dexamethasone (len/dex), with evidence of biochemical relapse or progression without new CRAB criteria (hypocalcemia, renal impairment, anemia, bone lesions), in whom cyclophosphamide was added at a dose of 50 mg on days 1–21 of a 28 day cycle. 10 patients achieved stable disease (SD), 6 a partial response (PR) and 3 a very good partial response (VGPR). Median OS was 18 months from the addition of cyclophosphamide and PFS was 13 months.^79^ Another center reported SD or better in 87% of 53 patients who had weekly cyclophosphamide, at a dose of 250–500 mg, added to len/dex at the time of progression.^80^

Cyclophosphamide also shows synergy with pomalidomide. A randomized phase 1/2 trial compared pomalidomide and dexamethasone (pom/dex) with or without 400 mg of weekly oral cyclophosphamide. Median PFS was 4.4 versus 9.5 months in favor or the cyclophosphamide arm without a statistically significant improvement in OS. 100% of the cohort were lenalidomide-refractory, and 75% were bortezomib-refractory, although prior exposure to pomalidomide was not discussed.^81^ 49 patients with prior exposure to lenalidomide and a PI were treated with pomalidomide, cyclophosphamide and dexamethasone in a single center retrospective, real-world, study. ORR was 76% with a median PFS of 7.3 months, which compared favorably with historical cohorts receiving pomalidomide/dexamethasone.^82^ For patients not responding adequately to pom/dex, the addition of cyclophosphamide was trialed in the single-arm phase 2 PERSPECTIVE trial. Following the addition of cyclophosphamide, of 16 patients with progressive disease on pom/dex, all patients achieved at least SD (3 PRs and 1 VGPR), and of 20 with either SD or minimal response, 45% responded with 5 patients achieving a PR, 2 and VGPR and 2 a CR.^83^ Results are awaited from a trial of cyclophosphamide, pomalidomide and dexamethasone versus pom/dex in patients with evidence of biochemical progression on lenalidomide maintenance (NCT03440411). Given that pomalidomide has enhanced immunomodulatory effects when compared with lenalidomide, the synergy with cyclophosphamide could possibly be greater. Low dose metronomic cyclophosphamide has very few side effects and is well tolerated by older, more frail patients. it offers a simple, inexpensive means of improving responses to IMiDs, even in those previously shown to be refractory.

### Monoclonal antibodies

The combination of the anti-CD38 monoclonal antibody, Daratumumab, with bortezomib and dexamethasone was compared with bortezomib/dexamethasone in RRMM in the phase 3 CASTOR trial. ORR was 83%, with VGPR or better in 59% and 19% of patients attaining CR.^84^ The addition of cyclophosphamide to this regimen has been shown to produce good outcomes in a multicenter non-randomized study (The LYRA study, NCT02951819). Most patients were newly diagnosed, and those who were refractory to a PI or PI/IMiD combination were excluded. Patients received 4–8 cycles, with the option to proceed to high-dose therapy with melphalan and autologous stem cell return. After induction, ORR and rate of VGPR or better were 81% and 56% respectively.^85^ A phase 1b clinical trial of daratumumab, with low-dose cyclophosphamide, bortezomib and dexamethasone in transplant-eligible NDMM (NCT02951819) reported ≥VGPR in 94% and ≥CR in 44%. Of 14 out of 15 patients who underwent ASCT and were evaluable for response, 57% achieved CR, and 83% in whom MRD could be assessed were negative to a sensitivity of 10^–5^ by next-generation sequencing.^86^ Enhanced macrophage-mediated ADCP was observed when MM cells were exposed to macrophages from these patients, suggesting a mechanism for the synergy seen, and providing a rationale for the incorporation of cyclophosphamide into similar treatment regimens.

Recently, the combination of daratumumab, low-dose dexamethasone, once weekly cyclophosphamide at 400 mg, with or without pomalidomide (DCdP vs. DCd), was tested in a randomized phase 2 clinical study in 120 patients with prior exposure to lenalidomide and a proteasome inhibitor. Cyclophosphamide enhances the activity of both monoclonal antibodies and IMiDs, so combination therapy is a logical approach. ORR was 88.5% vs. 50.8% in favor of the quadruplet, with ≥VGPR achieved in 57.4% vs. 25.4%. Median PFS was 10.9 months in the DCd arm and has not yet been reached for DCdP patients.^87^ Although DCd showed an inferior response rate at 50.8%, this is considerably higher than results seen with single-agent daratumumab, when tested in a relatively similar patient population.^88^

The observation that high doses of cyclophosphamide induce immunosuppressive MDSCs, whereas low doses do not should also be borne in mind. MDSCs inhibit macrophage activity through PGE2 secretion. They also inhibit NK cytotoxicity, production of IFN-gamma, and expression of NKG2D.^66^ Metronomic dosing of cyclophosphamide may therefore synergize more effectively with monoclonal antibody therapies than standard doses, by enhancing macrophage-mediated ADCP and avoiding suppression of NK-mediated ADCC. Several groups are developing methods to improve NK function in patients receiving monoclonal antibodies, such as administering infusions of engineered NK cells with low levels of CD38 expression to patients receiving daratumumab, which causes fratricide of CD38 expressing NK cells alongside the malignant MM cells.^89,90^ The addition of cyclophosphamide to such treatments could further improve responses and patient outcomes by ameliorating the immune suppression induced by the TME.

Cyclophosphamide has also been shown to induce SLAM-F7 expression on MM cells. A small phase 2 study of the SLAM-F7-targeted monoclonal antibody, elotuzumab in combination with thalidomide and dexamethasone in RRMM (n = 51) permitted the addition of metronomic cyclophosphamide to treatment in patients not achieving an adequate response after 4 cycles of therapy (n = 11). 51 patients were enrolled of which 11 received cyclophosphamide. This was a heavily treated, refractory cohort. The ORR was 38% with an OS of 16.3 months, which was not affected by the addition of cyclophosphamide, however the number of study participants was very small. Further investigation of this combination could be considered.^91^

### Cellular therapies

Animal models and some early phase studies have shown that cyclophosphamide can enhance the anti-tumor activity of adoptive T cell populations and tumor vaccines in the setting of various cancers, providing potential use in the field of adoptive cellular therapies.^52,92–96^ These effects are thought to be a consequence of reduced numbers of Tregs, and increased Th1 activity, inducing a state of relative immunopotentiation.^4,97^ One group showed that tumor-immune cells, isolated from vaccinated donor mice, migrated preferentially to tumor sites after adoptive transfer into a murine model of melanoma. This was only seen in mice which had been pre-treated with cyclophosphamide compared with non-cyclophosphamide exposed mice. The best anti-tumor responses were seen in association with increased levels the proinflammatory cytokines IL-7, IL-15, IL-2, IL-21 and IFN-gamma, which occurred during recovery following lymphodepletion.^98^

With respect to patients with MM, cyclophosphamide has been utilized as lymphodepletion either alone, or in combination with other agents, prior to administration of CAR-T cell (chimeric antigen receptor) therapies. An ongoing phase 1 study using LCAR-B38 M, a CAR-T cell therapy targeted against BCMA (B-cell maturation antigen) published results on 57 patients with RRMM who received lymphodepletion with 300 mg/m^2^ single-agent cyclophosphamide in 3 split doses, prior to CAR-T cell infusion. ORR was 88% with a median PFS of 15 months reported.^99^ Another CAR-T targeting BCMA was tested at 2 doses (1–5 × 10^7^ or 10^8^ CAR-Ts) with or without cyclophosphamide 1.5 g/m^2^ conditioning. At the higher cell therapy dose, initial results showed responses in 6 of 9 patients receiving the CAR-Ts alone and 5 out of 6 in conjunction with cyclophosphamide. Moreover, median peak expansion of the CAR-T product, measured by qualitative polymerase chain reaction (qPCR) was 6160, 14,761, and 45,268 copies/μg DNA for 1–5 × 10^8^ CAR-Ts, 1–5 × 10^7^ CAR-Ts with cyclophosphamide and 1–5 × 10^8^ CAR-Ts with cyclophosphamide respectively, indicating enhanced expansion of the adoptive cellular product following cyclophosphamide conditioning.^100^ Other groups have conditioned patients with cyclophosphamide at varying doses (from 3 infusions of 250 mg/m^2^ to 3 infusions of 1 g/m^2^) with fludarabine (3 infusions of 25–30 mg/m^2^).^101–104^

Whereas most CAR-T cell therapy studies have employed moderate-high doses of cyclophosphamide, a phase 1 trial of a kappa chain-directed CAR-T in patients with refractory non-Hodgkin lymphoma (NHL) or MM gave only 12.5 mg/kg cyclophosphamide to patients without inducing lymphopenia. Of 7 MM patients, 4 attained SD lasting 2–17 months.^105^ Although there is limited evidence, it may be possible that lower doses of cyclophosphamide are sufficient to potentiate persistence and enhanced activity of adoptive cellular products. Lymphodepletion is thought to support persistence of cellular therapies by suppressing Treg numbers, leading to higher levels of proinflammatory cytokines.^106^ Therefore, there may not be a requirement for absolute lymphopenia itself, but rather an alteration in the composition of the TME. Moreover, 4th generation CAR-T cells, or so-called armored CARs or TRUCKs can be engineered to constitutively express the relevant cytokines required to optimize persistence and efficacy of the cellular product *in vivo*, potentially obviating the need for lymphodepletion.^107^ There are currently no TRUCKs under investigation in MM, but this area warrants further study.

Enhancement of DC activity is also under investigation in MM. In a phase 1 clinical trial in RRMM, 2 doses of a DC vaccine loaded with autologous MM cells were administered weekly for 4 weeks to patients who had received prior thalidomide- and bortezomib-based regimens. The vaccine was well tolerated. Of 9 patients receiving the higher dose, 1 patient had a minor response, 5 had SD and 3 progressed.^108^ This study did not incorporate cyclophosphamide, however given the evidence that cyclophosphamide enhances dendritic cell antigen presentation and IL-12 secretion,^45^ this strategy could improve responses. Outside of the field of MM, the addition of a single 300 mg/m^2^ dose of cyclophosphamide prior to administration of a multipeptide vaccine in renal cell cancer lead to a reduction in peripheral blood Tregs and improved overall survival (23.5 months vs. 14.8 months, p = 0.09).^109^ The multivalent WT1 (wilms tumor 1) vaccine, Galinpepimut-S, was administered to poor cytogenetic risk myeloma patients alongside lenalidomide maintenance after high dose therapy and autologous stem cell transplant. An encouraging median PFS of 23.6 months was reported, leading to the designation of orphan drug status by the European Medicines Agency in 2018.^110^ Low dose cyclophosphamide could potentially augment this response through additional Treg inhibition.

## Conclusions and future considerations

Cyclophosphamide has played an integral role in the treatment of multiple myeloma for the best part of 50 years, as an alkylating agent and due to its ability to mobilize hematopoietic stem cells from the bone marrow. Its immunomodulatory properties enable synergistic responses with several classes of anti-myeloma therapies including the immunomodulatory agents, monoclonal antibodies and cellular therapies. As a consequence, the role of cyclophosphamide within modern myeloma therapy is changing and developing.

The monoclonal antibody Daratumumab has been approved for use in the frontline setting in non-transplant-eligible patients following results from the phase 3 ALCYONE trial, and is likely to receive approval for transplant-eligible patients as a consequence of the recent CASSIOPEIA trial.^111,112^ Low dose cyclophosphamide augments macrophage-mediated ADCP and NK-mediated ADCC, without inducing immune-suppressive MDSCs, and could usefully be added to monoclonal antibody-based regimens in newly diagnosed or relapsed refractory patients. Immune function progressively declines with length of disease course. The additional immunomodulation may therefore be of greatest benefit to patients who have had the disease for longer, in whom immune dysfunction is be more pronounced.

Cyclophosphamide synergizes with the IMiD drugs lenalidomide and pomalidomide producing meaningful responses in patients with documented resistance to these agents. The mechanisms underpinning this are not well characterized, but increased levels of tumor-specific activated T cells were demonstrated in patients receiving combination therapy with lenalidomide and cyclophosphamide in the REPEAT study,^78^ and may partly explain this observation. Various strategies combine monoclonal antibodies with IMiDs. Adding cyclophosphamide at low doses to such protocols could therefore potentiate activity of both the antibody and IMiD components through interactions with the TME, as suggested by early results from the phase 2 trial of daratumumab with cyclophosphamide, dexamethasone and pomalidomide.^87^ Low-dose cyclophosphamide is a well-tolerated oral option, which is of importance in heavily treated older patients, who may already have therapy-related sequelae, and in whom quality of life requires particular consideration.

Finally, possible potentiation of the efficacy of cellular therapies requires further investigation as products such as CAR-Ts and tumor vaccines continue to be developed and improved. High doses of cyclophosphamide are currently administered prior to infusion of CAR-T cells to induce lymphodepletion. Whether lower doses could induce the immunomodulation required to facilitate sustained persistence and activity of cellular therapies whilst avoiding the toxicities associated with lymphopenia, particularly in the context of cytokine-expressing 4th generation TRUCKs, has yet to be established.

Several important questions remain:

1. Which combinations of therapies produce the best synergism and at what doses?
2. When is the optimal time for cyclophosphamide-containing regimens to be used?
3. How can cyclophosphamide be utilized in the setting of cellular therapies?

We hope that future well-designed research can clarify these questions.
